# Mechanisms of learning and plasticity in childhood and adolescence

**DOI:** 10.1016/j.dcn.2020.100764

**Published:** 2020-01-30

**Authors:** Yana Fandakova, Catherine A. Hartley

**Affiliations:** Center for Lifespan Psychology, Max Planck Institute for Human Development, Berlin, Germany; Department of Psychology and Center for Neural Science, New York University

The study of child development is intricately linked to understanding the mechanisms of learning and plasticity through which experience gives rise to changes in brain and behavior. Through learning, the skills and knowledge that inform an individual’s behavioral repertoire are acquired and updated on the basis of one’s experience in changing environments. This learning process, at different time scales and across different stages of development, is accompanied by lasting changes in the developing brain. Plasticity, or the capacity of the brain to exhibit persistent structural and functional change, can take different forms, including the formation and elimination of synaptic connections, the modification of synaptic weights as well as the reorganization of brain networks and connections (Zatorre et al., 2012). Neural plasticity in turn, plays a critical role in shaping cognitive and behavioral developmental processes across childhood and beyond.

A useful framework that links learning, development, and plasticity distinguishes between experience-expectant and experience-dependent processes (Galván, 2010; Greenough et al., 1987). Experience-expectant processes denote cases in which the neural system has evolved to expect specific environmental inputs during particular time windows in development. During such sensitive periods, expected inputs are presumed to have increased impact on brain organization in ways that are largely shared across individuals (Greenough et al., 1987). In contrast, experience-dependent processes denote lasting neural changes resulting from experiences and environmental inputs that can vary considerably across individuals, and in the time windows in which they occur.

What are the mechanisms of learning and development? How does plasticity within specific neural circuits change across childhood and adolescence? How are sensitive periods in plasticity modulated by environmental influences? These questions were among the topics discussed at the Flux Congress in Berlin in 2018. The present collection of articles discusses recent empirical and theoretical advances in the study of learning and plasticity across childhood and adolescence. The breadth of methodological and analytical approaches reflected in this issue demonstrates how quickly the field is gaining a better understanding of how experience, learning, and maturation mutually influence each other throughout ontogeny, resulting in unique individual trajectories of development.

In an introduction to the special issue, Uta Frith reflects on progress that the field of developmental cognitive neuroscience has made for understanding learning and plasticity, along with the challenges that it is currently facing (Frith, 2019). While the field has successfully begun to identify brain regions that are sensitive to specific types of information during development and exhibit plasticity in function and structure in response to specialized training, Frith also points out a number of open questions that require continued research. These include mapping the trajectory and timeline of typical neurocognitive development, uncovering the limits of learning-dependent brain malleability, and elucidating the mechanisms of abnormal brain development. Below, we detail the central findings from the articles in this special issue, which address these fundamental research areas.

## 1 Typical and atypical learning and development

Over the past years, an increasing number of studies have started relating the development of learning across different domains to the development of underlying brain regions and networks. The quest to chart the timeline of brain and cognitive development has been further advanced by the use of new methodological approaches, including diverse brain imaging techniques, sometimes used in combination, the availability of large cross-sectional and longitudinal samples, and advanced methods for data analysis.

In this issue, Pleisch et al. used simultaneous EEG and fMRI to examine how functional language networks are altered in children with poor reading development within the first months of formal reading instruction (Pleisch et al., 2019). After about half a year of schooling, children at varying risk for developmental dyslexia showed coarse orthographic sensitivity in the N1 ERP component and in BOLD response in the ventral occipitotemporal cortex (vOT). Notably, the authors demonstrated that combining different neuroimaging methods increased sensitivity to capture small but meaningful functional differences in language networks in the developing brain. More specifically, combining ERP and fMRI measures within the left vOT showed that BOLD modulation in the left vOT by the N1 amplitude was stronger for words than false font strings in typical, but not in poor reading children, indicating more advanced orthographic tuning in the former group. Additionally, this work showed that vOT areas with preferential activation to print categories could only be captured when taking individual differences in vOT area location into account, thereby emphasizing the importance of considering individual differences in brain development and using methods that are sensitive to those differences.

The importance of considering individual differences in learning and their neural underpinnings are further highlighted by Iuculano et al. who examined how measures of numerical abilities are related to underlying brain mechanisms in children with autism spectrum disorder (ASD) and their typically developing peers (Iuculano et al., 2020). Typically-developing children showed a negative correlation between numerical abilities and functional brain activation in a network of brain regions associated with numerical cognition, whereas children with ASD showed the opposite effect. ASD children required greater amount of information to reach a decision, and this enhanced decision threshold moderated the relationship between individual differences in numerical abilities and engagement of prefrontal control systems. Turning to individual differences in learning during a short math tutoring program, Chang et al. demonstrated that while on average children improved performance with training of specific math problems, learning rates varied considerably across children (Chang et al., 2019). Specifically, faster learners during tutoring showed higher performance on both trained and novel math problems. On the neural level, faster learners showed a greater overlap of neural representations of trained and novel problems in medial-temporal, frontal and temporal regions, along with a reduced connectivity and greater segregation among the regions supporting math problem solving.

Studying a large longitudinal cohort of children and adolescents (4–25 years), Fjell et al. examined memory recall ability over development when tested at shorter or at extended retention intervals (Fjell et al., 2019). Recall after 10 min and after approximately 30 min showed distinct developmental trajectories. For verbal memory, short and extended retention displayed similar development until around 10 years of age, whereas between 10 and 15 years of age, improvements in extended retention performance exceeded the increases in shorter delay performance. For visual-spatial memory, there were linear increases in extended retention performance across the age range beyond improvements in shorter delay performance. In addition, higher recall performance across time intervals and tasks was associated with microstructure of the anterior hippocampus and with lower lateral prefrontal thickness. In a longitudinal sample of 12–26 year-old participants, Ferschmann et al. focused on the neural underpinnings of prosocial behavior development during adolescence (Ferschmann et al., 2019). The authors showed that in regions associated with social cognition and behavioral control, higher self-reported prosociality was related to greater cortical thinning during early-to-middle adolescence, followed by attenuation of this process during the transition to young adulthood. In contrast, lower prosociality was related to initially slower thinning, followed by comparatively protracted thinning into young adulthood.

In two cross-sectional child and adolescent samples, Simpson-Kent et al. used structural equation modeling to examine the factorial structure and neural substrates of intelligence (Simpson-Kent et al., 2020). Cognitive ability in lower- and typical-ability cohorts was best described by two separable constructs, crystallized and fluid intelligence, which became more distinct with age. White matter microstructure, most prominently the superior longitudinal fasciculus, was strongly associated with crystallized and fluid abilities. Notably, the relationships between crystallized and fluid abilities, and their white matter substrates differed dynamically by age, such that they dropped between 7–8 years before increasing around age 10. Together, this study highlights the importance of the phase shortly before puberty for the development of the neurocognitive architecture of intelligence. The neural mechanisms supporting cognitive development are further investigated by Marek et al. using data from the ABCD study (Marek et al., 2019). The authors examined resting-state functional connectivity and its relations to general cognitive ability across demographically-matched discovery and replication datasets of 9-10-year old children. Resting-state connectivity and network architecture were highly reproducible across children. In addition, a widely-distributed circuitry, including connectivity within and between several functional networks, was associated with higher cognitive ability. Notably, the authors highlighted a number of important sources of variation in the results. In particular, scanner manufacturer effects were relatively large, reproducible, and followed a “short-to-long” association with distance between regions. Future years of the ABCD study will be able to precisely characterize maturational resting-state profiles in a longitudinal fashion, providing a powerful resource for normative adolescent growth curves of resting-state functional connectivity.

## 2 Learning systems

In recent years, a large literature has characterized multiple dissociable learning systems in the adult brain. Several studies in this issue extend this body of research across development, elucidating the neurocognitive underpinnings of these systems and the nature of their interactions in childhood or adolescence.

Werchan and Amso conducted a fNIRS study to investigate how 9-month-old infants learn which competing features of a cluttered visual environment are informative for subsequent attention in novel contexts (Werchan and Amso, 2020). Infants rapidly acquired top-down knowledge of relevant visual features through abstract rule learning. They also successfully generalized the rule to modulate visual attention to learned behaviorally-relevant visual features in a novel situation. Notably, infants who showed better rule generalization ability also demonstrated greater connectivity between PFC and visual cortex.

In a cohort of adolescents aged 13–20, Insel et al. examined developmental changes in the interaction between value-based learning and cognitive control systems (Insel et al., 2019). In adults, cues previously associated with high value outcomes have been found to enhance subsequent goal-directed behavior. Here, the authors sought to chart the developmental trajectory of this effect. The beneficial effect of learned value associations on subsequent cognitive control performance was observed to emerge with age, and was associated with differences in the recruitment of corticostriatal circuitry. These findings highlight the importance of elucidating how changing interactions between cognitive processes alter learning across development. Master et al. also focus on characterizing the development of interactive cognitive mechanisms that support learning, examining the individual contributions of reinforcement learning and working memory to learning in childhood and adolescence, (Master et al., 2020). Children and adolescents (8–17 years) and adults learned stimulus-action associations from feedback with a learning load that was varied to either be within or to exceed working memory capacity. Participants aged 8–12 years learned more slowly than participants aged 13–17 years, and were more sensitive to working memory load. Using computational modeling to estimate use of working memory and reinforcement learning processes across participants, the authors observed more robust age differences in reinforcement learning than in working memory. Reinforcement learning rate increased significantly with age across adolescence, whereas working memory parameters showed more subtle changes, many of them early in adolescence. These results underscore the importance of changes in reinforcement learning processes for learning in childhood and adolescence. This point is further emphasized in the review paper by Nussenbaum and Hartley who offer an insightful summary of how our understanding of the development of value-based learning has been advanced by using reinforcement learning models to examine age differences (Nussenbaum and Hartley, 2019). Across paradigms and age groups, the available evidence suggests that individuals become better at optimally weighting recent outcomes during learning across diverse contexts and less exploratory in their value-based decision-making.

## 3 Sensitive periods

It is now well established that development is characterized by time windows of increased sensitivity to specific environmental inputs (Knudsen, 2004). These sensitive periods of increased plasticity in brain development have been well documented with respect to different functions, for example for sensory systems (Banks et al., 1975) or language (Werker and Hensch, 2015), and differ markedly with respect to their timing across development.

In this issue, Pant et al. examined plasticity in cortical areas typically associated with the processing of visual information in congenital blindness, where these areas begin responding to linguistic information (Pant et al., 2020). The authors compared the neural basis of sentence processing between adult-onset blind, congenitally blind and blindfolded sighted adults to investigate whether these changes follow a sensitive period. While the visual cortices of congenitally blind and adult-onset blind individuals responded more to sentences than control conditions, the effect was much larger in the congenitally blind who were the only group in which visual cortices responded to grammatical complexity. These results suggest that blindness during a sensitive developmental period modifies the neural basis of language. Moreover, recent years have seen a profound progress in understanding the neural mechanisms controlling the timing of these sensitive periods (Hensch, 2004). Adopting an evolutionary perspective, Frankenhuis and Walasek highlight progress in understanding the evolution of sensitive periods (Frankenhuis and Walasek, 2020). They discuss the central tenets, insights, and predictions of mathematical models, in relation to empirical work on humans and other animals. The authors propose future models which are needed to improve the bridge between theory and data, advancing their synergy.

While sensitive periods in early childhood have been demonstrated for different functions, the extent to which adolescence represents a sensitive period and for what is a matter of ongoing discussion. Starting from the idea that adolescence may be a sensitive period for social development, with a particularly strong negative influence of social exclusion on brain and behavior, Fuhrmann et al. set out to examine how social exclusion influences cognitive performance in adolescent girls (Fuhrmann et al., 2019). While younger and older adolescents as well as adults showed reduced mood following a brief exposure to social exclusion, only younger adolescents showed reduced verbal working memory performance. These results suggest that the social environment may be particularly important in early adolescence when negative effects of exclusion have more widespread effects and negatively influence cognitive performance. Turning to higher-order cognitive abilities such as memory and executive functioning, Laube et al. discuss the potential role of pubertal hormones for regulating sensitive periods in these abilities (Laube et al., 2020). The authors review animal and human brain imaging studies, which indicate that pubertal hormones play a pivotal role in regulating the mechanisms of experience-dependent plasticity during adolescence. However, the extent to which hormonal changes associated with pubertal onset increase or decrease brain plasticity may depend on an individual’s sex, the specific cognitive domain in question, and its associated brain networks.

## 4 Environmental influences

Environmental factors play an important role for learning in development. The effects of particular external changes in the environment may be particularly high during sensitive periods, in which children and adolescents show increased sensitivity to specific environmental inputs (Andersen and Teicher, 2008). Socio-economic status (SES) is one factor that has received considerable attention in terms of its effects on learning and development. Low SES has been associated with lower performance across multiple cognitive domains, including memory and executive functioning (Noble et al., 2007), and has also been related to differences in brain development (Sheridan et al., 2012). However, SES reflects a myriad of differences in a child’s environment that may contribute to cognitive and brain development in different ways.

In this issue, Rosen et al. investigated the environmental factors that contribute to SES disparities in cognitive performance and academic achievement in early childhood, a period marked by increased experience-dependent plasticity (Rosen et al., 2019). Specifically, they examined how violence exposure, cognitive stimulation, and the quality of the physical environment were associated with memory, attention and memory-guided attention. Violence exposure was associated with lower memory performance, whereas higher quality of the physical environment was related to better memory-guided attention. In a longitudinal sample of 6-to-7 year-olds, Raffington et al. found that lower family income was associated with poorer memory performance and smaller hippocampal volume across middle childhood, and these associations remained stable over time (Raffington et al., 2019). While polygenic scores of educational attainment were associated with family income, genetic variance captured by the polygenic scores did not moderate the relationships of income with hippocampal volume and memory. Together, these studies suggest that specific aspects of early environmental experiences that are distinct from genetically-mediated transactional pathways contribute to individual differences in different cognitive functions and their corresponding neural underpinnings.

Animal models are particularly well suited to gain further insights into the specific mechanisms by which various environmental factors affect neural development. Perry et al. examined the biological mechanisms by which poverty-related adversities influence social behavior (Perry et al., 2019). Using a rodent model of scarcity (i.e., material resource deprivation) and adversity (i.e., reduced caregiving quality), they explored how early-life exposure to these environmental conditions causally influences social behavior via disruption of developing stress physiology. In rodents, early-life exposure to scarcity via material resource deprivation in combination with exposure to adversity via reduced caregiving quality lead to increased social avoidance in peri-adolescence. These behavioral changes were accompanied by blunted hypothalamic-pituitary-adrenal (HPA) axis activity and elevated glucocorticoid receptor levels in the dorsal hippocampus and medial prefrontal cortex. Notably, administration of corticosterone rescued social behavior, providing support for the role of glucocorticoids in the negative effects of early-life scarcity and adversity on later social development. Finally, Thomas et al. examined how early-life adversity, in particular maternal separation, may affect the development of synaptic density on long range frontal cortex projections (Thomas et al., 2020). Maternal separation and variation in maternal care predicted bouton density on dorsal frontal cortex axons that terminated in the basolateral amygdala at mid-adolescence postnatal day 35 and axons that terminated in dorsomedial striatum in adulthood at postnatal day 85. In both cases more, fragmented care was associated with higher density, suggesting that early-life adversity can alter development in a circuit-specific and age-dependent manner.

## 5 Future directions

This Special Issue features a wide range of studies that examine different aspects of learning and plasticity in childhood and adolescence. These contributions showcase the timely questions that currently dominate the field, and the cutting-edge methodological techniques and experimental approaches used to examine the mechanisms of learning and plasticity. Going forward, there are continued opportunities for methodological innovation and conceptual advances in these research areas, many of which are raised in Uta Frith’s introductory article.

As the numbers of longitudinal data sets are increasing and methods for analyses are becoming more sophisticated, we need to find successful ways to examine causal relationships between specific aspects of experience and neurocognitive differences, in order to more clearly delineate the mechanisms that underlie observed developmental change. Frith suggests that tracking children with neurodevelopmental disorders may be a helpful approach to incorporate in this endeavor. Furthermore, combining animal and human studies is likely to play an essential role for better understanding sensitive periods and specific environmental effects in development. Frith also emphasizes the need for precise methods and adequate statistical power in order to ensure the robustness of findings. Frith encourages researchers to be open to asking the hard questions, including those about individual differences in learning and plasticity. Finally, Frith underscores the importance of conducting research that is informed by good theories that parsimoniously integrate existing research findings and advance our understanding of the dynamic mechanisms of learning and plasticity that underpin neurocognitive development.
